# Thrombophlébite cérébrale inhabituelle du post-partum

**DOI:** 10.11604/pamj.2014.18.318.5014

**Published:** 2014-08-21

**Authors:** Moulay Elmehdi El Hassani, Abdellah Baba Habib, Jaouad Kouach, Youssef Sekkach, Hicham Bakkali, Mohamed Jira, Mohamed Elqatni, Ali Abouzahir, Dris Ghafir, Dris Moussaoui, Mohamed Dehayni

**Affiliations:** 1 Service de Gynécologie-Obstétrique, Hôpital Militaire d'Instruction Med-V, Rabat, Maroc; 2 Département de Médecine Interne-B, Hôpital Militaire d'Instruction Med-V, Rabat, Maroc; 3 Service de Réanimation Anesthésie, Hôpital Militaire d'Instruction Med-V, Rabat, Maroc

**Keywords:** anticoagulants, MRI, post-partum, thrombophlébite cerebrale, Anticoagulants, IRM, post-partum, cerebral thrombophlebitis

## Abstract

La thrombophlébite cérébrale du post-partum immédiat constitue un événement rare et gravissime pouvant mettre en jeu le pronostic vital à court terme. Celle-ci doit être systématiquement évoquée devant la persistance d'une fièvre dans les suites de couches. La prise en charge associera le plus souvent, des antibiotiques à large spectre et des anticoagulants. Le suivi évolutif est indispensable, afin d'apprécier l'efficacité thérapeutique. A travers une observation singulière et à présentation inhabituelle, nous insistons sur le grand intérêt des moyens d'imagerie dont nous disposons afin de porter le diagnostic et de choisir le traitement le mieux adapté.

## Introduction

La thrombophlébite cérébrale du post partum est une entité rare mais sa survenue est grave et peut compromettre le pronostic vital. Elle représente 10 à 20% des TVC [1]. La grossesse est le post partum sont des situations à risque du fait de l'hyper-coagulabilité physiologique qui les accompagne. Le tableau clinique est variable en fonction da la topographie des thromboses, ce qui rend le diagnostic précoce difficile. L'imagerie et en particulier l'angio IRM est l'examen de référence qui permet un diagnostic précoce. L’évolution est généralement favorable sous traitement bien conduit. A travers une observation de TPC du post partum à présentation inhabituelle nous essayons de montrer l'intérêt des moyens d'imagerie dans le diagnostic précis de la TPC, afin de proposer le traitement adéquat.

## Patient et observation

Mme A. R âgée de 30 ans, multipare, sans antécédent susceptible et n'ayant jamais reçu de traitement œstroprogestatif. Elle a accouché par les voies naturelles au terme d'une 3ème grossesse normale et menée à terme. Des céphalées posturales diffuses et une ophtalmoplégie hyperalgique associés à des cervicalgies inflammatoires et une fièvre, apparaissaient dans le post-partum immédiat (j4).

L'examen clinique à l'admission retrouvait une patiente fébrile à 39,7°, consciente et bien orientée dans le temps et dans l'espace. La nuque était raide à la tentative de mobilisation, avec une douleur projetée en regard des deux régions parotidiennes. L'attention était également attirée par une exophtalmie bilatérale, avec un œdème palpébral sans goitre. Le bilan biologique retrouvait une hyperleucocytose à 14900/mm3 à prédominance neutrophile, sans anémie ni thrombopénie, de même qu'un important syndrome inflammatoire (CRP à 230mg/l) et un bilan thyroïdien normal. Tous les prélèvements à visée infectieuse (hémocultures, prélèvement de gorge, ECBU, sérologies des hépatites virales B, C et VIH, de même que les sérologies d'EBV, CMV, Herpes et parvoB19) étaient négatifs, de même que le bilan immunologique (Ac anti-nucléaires, Ac anti-phospholipides). La persistance et l'exacerbation des douleurs et de la fièvre, amenait à la réalisation d'un scanner cérébral sans injection couplé à un scanner cervico-thoraco-abdomino-pelvien avec injection qui étaient respectivement sans anomalies.

**Figure 1:**
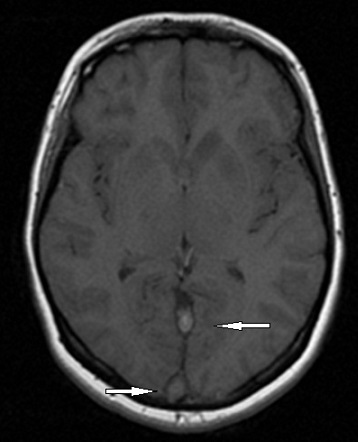
IRM cérébrale en coupe transversale (T1) sans injection montrant la thrombose des sinus droit et longitudinal supérieur

**Figure 2:**
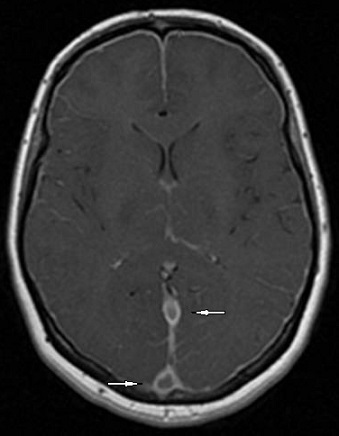
IRM cérébrale en coupe transversale avec injection de gadolinium montrant la thrombose des sinus droit et longitudinal supérieur apparaissant en hyper signal T2

**Figure 3:**
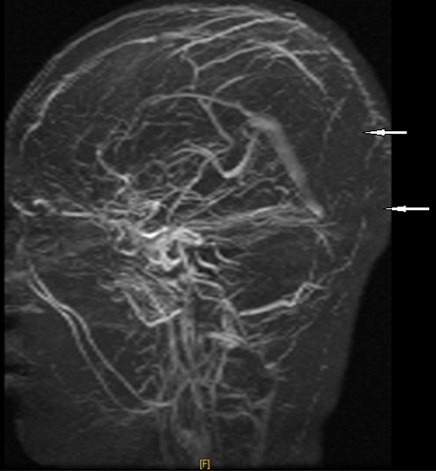
Séquence d'angio IRM veineuse: la non visibilité des sinus droit et longitudinal supérieur

**Figure 4:**
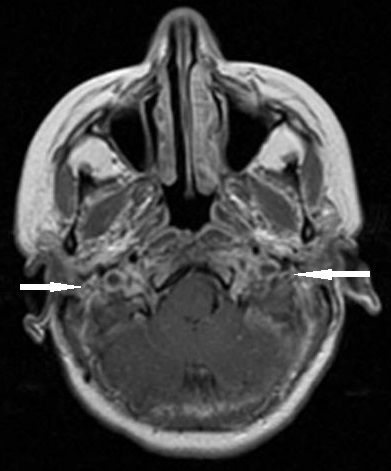
Coupe transverse en T1 avec gadolinium montrant l'extension de la thrombose au golf des veines jugulaires

A la ponction lombaire, le liquide était clair « eau de roche », avec une très faible céllularité (8 GB/mm^3^ et 70GR/mm^3^), une protéinorrachie et une glycorrachie normales, de même qu'une culture stérile et une recherche d'antigènes solubles négative. Le tableau clinique évoluait à j10 vers un tableau d'hypertension intracrânienne associée à un œdème papillaire au fond d’œi1. L'IRM cérébrale ainsi réalisée mettait en évidence, en plus d'un œdème cérébral diffus et une exophtalmie bilatérale grade II, la présence de deux lésions lenticulaires et de la tête du noyau caudé en hypo signal T1 et en hyper signal T2, FLAIR des sinus, droit et longitudinal supérieur (Figure 1) prenant le contraste en périphérie après injection de Gadolinium (Figure 2), avec individualisation des thrombus en leur sein et développement d'un important réseau de suppléance (Figure 3). Cet aspect IRM était compatible avec une thrombophlébite cérébrale étendue jusqu'au golfe des jugulaires (Figure 4). Après un bolus IVD de 50UI/kg, un traitement par héparine IV, non fractionnée, était entrepris en continu, à la seringue électrique à la dose de 500 UI/kg/jour (25 000UI/24 h) associée à du Solumédrol en flash de 120 mg/jour pendant trois jours, permettait une amélioration très nette de la symptomatologie dès les 1ères 24 heures. Le fond d’œil s’était normalisé et les signes radiologiques régressaient plus lentement et n’étaient plus constatés à j45.

Un traitement par Anti-vitamine K (Sintrom 4mg) fut prescrit en relais de l'héparinothérapie à j21 pendant 6 mois. La négativité du bilan étiologique faisait ainsi retenir comme seul facteur causal à cette thrombophlébite cérébrale «le post-partum». Le bilan de thrombophilie (protéine C, protéine S, antithrombine III, facteur V de leyden ainsi que la résistance à la protéine C), réalisé 3 semaines après l'arrêt des anticoagulants, ne révélait aucun déficit.

## Discussion

L'incidence moyenne des thrombophlébites cérébrales (TVC) au cours de la grossesse et du post-partum est de 15 à 20 pour 100 000 accouchements, ce qui représente 10 à 20% des TVC [1]. Elles surviennent le plus souvent au décours d'un accouchement normal et jusqu’à dix semaines après l'accouchement, avec un pic de fréquence entre le 4è et 21è jour comme le cas pour notre patiente. Cependant, il existe des cas plus précoces et d'autres beaucoup plus tardifs [1]. Bien que la TVC puisse survenir à tout moment au cours de la vie, les femmes sont particulièrement vulnérables avant l′accouchement et pendant la période post-partum en raison de l′état d′hyper-coagulabilité qui accompagne la grossesse [2].

Les signes et symptômes peuvent varier en fonction de la topographie de la thrombose veineuse. Toutefois, la variation interindividuelle de l'anatomie veineuse cérébrale ainsi que la fréquente association de thrombose de plusieurs sinus et veines rendent difficile une corrélation clinico-topographique précise. L'atteinte du SLS (70%) et du SL (70%) est la plus fréquente, suivie de l'atteinte du sinus droit (15%) puis du sinus caverneux (3%). La thrombose du SLS et/ou d'un SL ou d'un sinus caverneux s'accompagne volontiers d'une association variable de symptômes.

Le diagnostic doit être d'emblée évoqué devant une combinaison variable des signes suivants: hypertension intracrânienne, déficit neurologique focal, crises épileptiques, chémosis, ophtalmoplégie douloureuse, exophtalmie, œdème palpébral, atteintes de nerfs crâniens. Les signes et symptômes initialement unilatéraux peuvent devenir bilatéraux si la thrombose s’étend aux autres sinus [3], comme le cas dans notre illustration. Dans les formes graves, les troubles de conscience peuvent évoluer vers le coma, associés à des troubles végétatifs [4]. Le diagnostic des TVC du post-partum (TVCP) repose sur l'imagerie. La TDM cérébrale demeure actuellement l'examen de débrouillage pratiqué en première intention. Elle montre rarement des signes directs [5].

Lorsqu'elle est disponible, l'IRM associée à l'angio-IRM veineuse est l'examen de référence pour le diagnostic précoce de TVCP [6, 7]. En IRM, le signal du thrombus dépend de son ancienneté et est particulièrement bien mis en évidence par la séquence T2 dans les 3 premiers jours. La présence d'une atteinte thalamique bilatérale en hyper signal T2 et FLAIR est fortement évocatrice du diagnostic. Toutefois, dans la TVCP, les lésions sont plus étendues, dépassant la portion médiale des thalami pour atteindre l'ensemble des noyaux gris centraux, réalisant un aspect en « ailes de papillon » [6]. Le diagnostic de TVCP peut être confirmé soit par un angioscanner ou idéalement par une angio-IRM après injection de gadolinium ou une angio-IRM veineuse de flux. L'IRM présente l'avantage par rapport la TDM d’être une technique non irradiante et ne nécessite pas d'injection d'agent de contraste en première intention pour affirmer l'occlusion veineuse [6, 7].

Considérées initialement d’évolution souvent fatale, ces thromboses, grâce aux progrès diagnostiques, sont de meilleur pronostic en permettant l'instauration d'un traitement anticoagulant précoce. L’évolution clinique est en général favorable avec récupération neurologique quasi-complète (80% des cas) [1]. Le traitement anticoagulant est maintenant largement recommandé dans le traitement des TVC documentées, et l'intérêt d'utiliser des thrombolytiques locaux n'est pas démontré, même dans les formes les plus aigües [1].

## Conclusion

La thrombophlébite cérébrale du post-partum est une complication qu'il faut savoir évoquer et rechercher. Malgré l'absence de consensus vis-à-vis de la durée optimale du traitement médicamenteux, la prise en charge associera le plus souvent, des antibiotiques à large spectre et des anticoagulants. Le suivi évolutif est indispensable, afin d'apprécier l'efficacité thérapeutique par la constatation de la disparition complète du phénomène thrombotique.
